# Griffiths phase and long-range correlations in a biologically motivated visual cortex model

**DOI:** 10.1038/srep29561

**Published:** 2016-07-20

**Authors:** M. Girardi-Schappo, G. S. Bortolotto, J. J. Gonsalves, L. T. Pinto, M. H. R. Tragtenberg

**Affiliations:** 1 Departamento de Física, Universidade Federal de Santa Catarina, 88040-900, Florianópolis, Santa Catarina, Brazil; 2 Departamento de Engenharia Química e Engenharia de Alimentos, Universidade Federal de Santa Catarina, 88040-900, Florianópolis, Santa Catarina, Brazil

## Abstract

Activity in the brain propagates as waves of firing neurons, namely avalanches. These waves’ size and duration distributions have been experimentally shown to display a stable power-law profile, long-range correlations and 1/*f *^*b*^ power spectrum *in vivo* and *in vitro*. We study an avalanching biologically motivated model of mammals visual cortex and find an extended critical-like region – a Griffiths phase – characterized by divergent susceptibility and zero order parameter. This phase lies close to the expected experimental value of the *excitatory postsynaptic potential* in the cortex suggesting that critical be-havior may be found in the visual system. Avalanches are not perfectly power-law distributed, but it is possible to collapse the distributions and define a cutoff avalanche size that diverges as the network size is increased inside the critical region. The avalanches present long-range correlations and 1/*f *^*b*^ power spectrum, matching experiments. The phase transition is analytically determined by a mean-field approximation.

The most important feature of a living complex system to survive is adaptability. In order to adapt, the organism cannot be inflexible, but also cannot act randomly. The border of chaos or a critical behavior seems to be the best evolutionary choice to survival beings^1^,^2^. Turing conjectured a similar idea referring to our minds and learning machines^3^.

More recently, brain criticality has become a trendy research subject^4^,^5^,^6^. Some believe that the brain became critical by self-organization and selection^4^, analogously to the sandpile self-organized criticality (SOC)^7^. Criticality has the advantages of maximizing the response dynamic range of neural networks^8^, optimizing memory and learning processes^9^, the computational power of the brain^10^ and information processing flexibility^11^.

The brain critical state is usually experimentally characterized via power-law (PL) distributed neuronal avalanches^12^,^13^,^14^,^15^,^16^ or diverging long-range spatial or temporal correlations^17^,^18^. On the other hand, criticality in (non-)equilibrium Statistical Mechanics is only rigorously defined via the PL convergence to zero of an order parameter and the simultaneous PL divergence of its associated susceptibility^19^,^20^,^21^. In addition, finite systems avalanche size distributions present cutoffs, which also must diverge according to PLs on the critical state^22^. These PL critical exponents must follow well-defined scaling relations^20^,^21^,^23^,^24^. Notice, however, that some authors do not take into account the finite-size scaling (FSS) of avalanche distributions cutoff^16^. Also, only a few authors use an order parameter-susceptibility pair to probe for criticality in Neuroscience^25^,^26^.

Here we show that there is a Griffiths^26^,^27^,^28^ phase (GP) in the non-equilibrium percolation-like phase transition of the visual processing activity as a function of the *excitatory postsynaptic potential* (EPSP). The GP is known to emerge from rare over-active region effects due to the quenched disorder of the network. One of the macroscopic consequences of such effects is the appearance of an extended region of critical behavior in the phase diagram of the system. This result for the visual system is consistent with recent findings that suggest that excitable systems running over the connectome structure (a whole brain network) present GP^26^. Our control parameter is the EPSP and the GP is found close to the experimental values of EPSP in the cortex^29^,^30^,^31^. The EPSP threshold for a complete network activation is determined via a mean-field approximation and is close to the expected numerical and experimental values of this parameter.

We define the density of activated neurons as an order parameter and verify that it converges to zero whereas its associated susceptibility diverges inside the GP by applying standard FSS technique. This scaling rigorously defines the critical phase transition in our model. Our order parameter is usual for absorbing state phase transitions^21^,^32^. Additionally, we show that the avalanches are PL distributed with a cutoff that scales with the system size inside the GP. We also study the visual system avalanches correlations and power spectrum. Throughout the critical region, the power spectrum of avalanche time series has the form 1/*f*^*b*^, with 0.2 ≤ *b* ≤ 1.3 as experimentally expected^17^,^33^,^34^,^35^,^36^. The activity time series inside the critical region also presents long-range correlations yielding *Detrended Fluctuation Analysis*^17^,^37^,^38^ (DFA) exponent 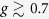.

Avalanche distributions presenting PL alone have been questioned as insufficient evidence for identifying the critical regime^10^,^39^,^40^ since there may be critical dynamical systems which have no PL distributed avalanches^39^ and non-critical dynamical systems that present PL distributed avalanches^40^. Our model’s avalanche distributions have PL shape inside the GP and even outside it. Thus, we show another example that PL distributed avalanches is not a sufficient condition for criticality. More than that, we use an order parameter-susceptibility pair as usually done for phase transitions to probe for criticality^20^,^21^,^23^,^24^.

We chose to model the visual cortex because it has a well-known anatomy and function^41^,^42^,^43^,^44^,^45^. The visual cortex also has a valuable data set available that we may use to benchmark our model results, such as the power spectrum^17^,^33^,^34^,^35^,^36^ and a few avalanche experiments^13^,^14^,^16^. We focus on understanding the dynamics of the signal propagation and the avalanche activity related to the network disordered structure. Our model is biologically motivated in the sense that the network we study here is layered, columnar, and recurrent, resembling the architecture of the visual cortex^45^ (full details of the network are given in the Supplementary Material). Also, our parameters are either fitted to experiments (such as the attenuation constant^29^) or have been experimentally measured (such as the structural parameters^41^,^42^,^43^,^44^).

The avalanches spontaneously emerge after a flash stimulus presented to the the model’s retina, instead of artificially imposing an abstract Poisson stimulus as in most of the brain critical models (see^8^,^9^,^13^,^16^,^46^,^47^,^48^,^49^ to cite a few). Avalanche dynamics is thus essential for the reliable signal propagation in our model.

We describe key features of the considered model in the following section. In the introduction of the Results section, we define the Griffiths phase and briefly discuss its origin in the model, the tools we use to characterize it (order parameter and susceptibility) and the observables of interest (avalanches, processing time, avalanche distributions and correlation measurements). Results are presented and discussed in the subsections of Results section. We finish the paper by briefly reviewing our main results and pointing how they relate to experiments and to SOC models in the Concluding Remarks section.

## Model

**Figure 1:**
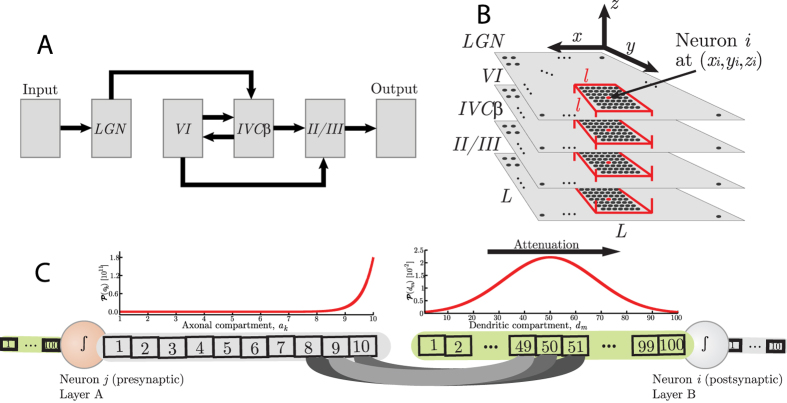
Elements of the V1 model. (**A**) Architecture of the network. (**B**) Spatial organization of the network of *N* = 4*L*^2^ neurons. The columnar structure is highlighted in red. There is a column of size *N*_*c*_ = 4*l*^2^ = 196 neurons centered on each neuron of the network. (**C**) Compartmental scheme of neurons. The probability, 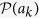, of choosing a presynaptic axonal compartment, *a*_*k*_, of any neuron is exponential such that most of the synapses start from the end of the axon (left). The probability, 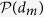, of choosing a dendritic postsynaptic compartment, *d*_*m*_, is Gaussian with mean 50 and standard deviation 10, so that most of the synapses lay in the middle of the dendrite (right).

This model was originally developed by Andreazza & Pinto^50^ in order to study the signal propagation dynamics in the visual cortex of mammals. They detected some sort of phase transition which will be analyzed in details throughout this work. The model is composed of six interconnected square layers. The signal propagates directionally from the retina (the Input layer) to the secondary visual cortex (Output layer). The other four internal layers have lateral size *L* and are selected from the form recognition pathway: the lateral geniculate nucleus (LGN, from the thalamus) and the layers II/III, IVC*β* and VI from the primary visual cortex (V1). The architecture of the network is illustrated in Fig. 1A, where arrows point the directions of the connections through which the signal propagates. The direction of the connections characterizes the adjacency of layers. The LGN layer consists of only its parvocellular neurons. Their synapses are mostly connected to V1 layer IVC*β*^41^,^42^,^43^,^44^. The synaptic buttons density over the dendrites is also based on experiments^41^,^42^,^43^,^44^. The bulk of the network (layers LGN, VI, IVC*β* and II/III together) has *N* = 4*L*^2^ neurons. The Input layer (composed of photoreceptors) and the Output layer (composed of axon terminals that connect to secondary visual cortex) have *N*_*io*_ = (10*L*)^2^ elements each. Each neuron *i* of the four internal layers is composed of a dendrite with 100 compartments, the soma, and an axon with 10 compartments.

The network is built in four steps: (a) for each neuron of the network, a postsynaptic neuron is chosen from an adjacent layer using a two-dimensional Gaussian distribution inside a limited excitatory field of size *l*^2^ in front of the presynaptic neuron; (b) an axonal compartment is chosen from the presynaptic neuron using an exponential probability distribution plotted in Fig. 1C (left); (c) a dendritic compartment of the postsynaptic neuron is chosen using a Gaussian distribution centered in the middle of the dendrite, as in Fig. 1C (right); and (d) a synapse is formed by connecting the chosen axonal compartment and the chosen dendritic compartment. There is a different number of outward synapses per presynaptic neuron depending on the presynaptic layer (see tables in Supplementary Material). The randomly chosen postsynaptic neurons and the randomly chosen pairs of presynaptic axonal compartments and postsynaptic dendritic compartments give rise to quenched disorder.

This structure generates a directed path of square columns of highly connected neurons for the signal propagation centered in each neuron of each layer. Each column has *N*_*c*_ = 4*l*^2^ ≈ 200 neurons (see Fig. 1B). As an example, a network with *L* = 99 (the largest considered size in this work) has approximately *N* = 4 × 10^4^ neurons and 32.5 × 10^6^ synapses in total. It is thus computationally costly to simulate larger networks. Once the network structure is built, it is kept fixed for a single signal propagation dynamics.

The EPSP (in the dendrites) and the action potential (in the axons) advance one compartment per time step *t*, coming from the dendrites through the soma to the last axonal compartment. The variables 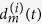 [Equation (1)], *v*_*i*_(*t*) [Equation (2)] and 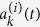 [Equation (3)] represent the local values of the EPSP sum (for each dendritic compartment *m*) or the action potential (for the soma and each axon compartment *k*) in the membrane of neuron *i* at time *t*, respectively.

These rules may be summarized in the following equations:


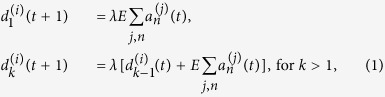



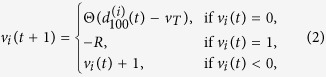



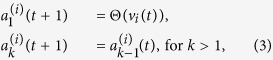


where *E* > 0 is the EPSP, *λ* = 0.996 is the dendritic attenuation constant (chosen to match experimental attenuation^29^), Θ(*x*) is the Heaviside step function and *v*_*T*_ = 10 mV is the firing threshold needed to induce an action potential^51^. After the soma spikes, it is reset to the value *R* which represents the refractory period (in time step unit). *R* is set to avoid self-sustained activity in the interlayer loops (between layers IVC*β* and VI). The signal propagates in the direction of increasing *k* (as *t* increases). The control parameter *E* sets the level of excitation, whereas *λ* sets the level of dissipation. The double sum in Equation (1) is over each of the axonal compartments *n* of the presynaptic neuron *j* connected to the dendritic compartment *k* of the postsynaptic neuron *i*.

Every neuron initial condition is given by 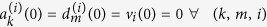. The time scale of the model is arbitrary, so the spike signal of the soma takes about 1 ms. The different number of compartments for dendrites and axons copes with their different velocities for the signal propagation^52^.

## Results and Discussion

For each *E* and *L*, the activity is initiated by flashing once a square of 30 × 30 photoreceptors in the center of the Input layer (which, in turn, will activate a region of 3 × 3 neurons in the LGN). We also consider different positions for the initial stimulus, such as flashing a square near the corner of the Input layer in order to verify if results are stable. And indeed they are notice that this procedure is similar to that used in the study of epidemic spreading in systems having absorbing phase transitions, in which a single site (or a small fraction of sites) is initially infected^21^. However, in epidemic spreading models each site is a simple three or four states cellular automaton and the network is generally regular and hypercubic^21^.

**Figure 2:**
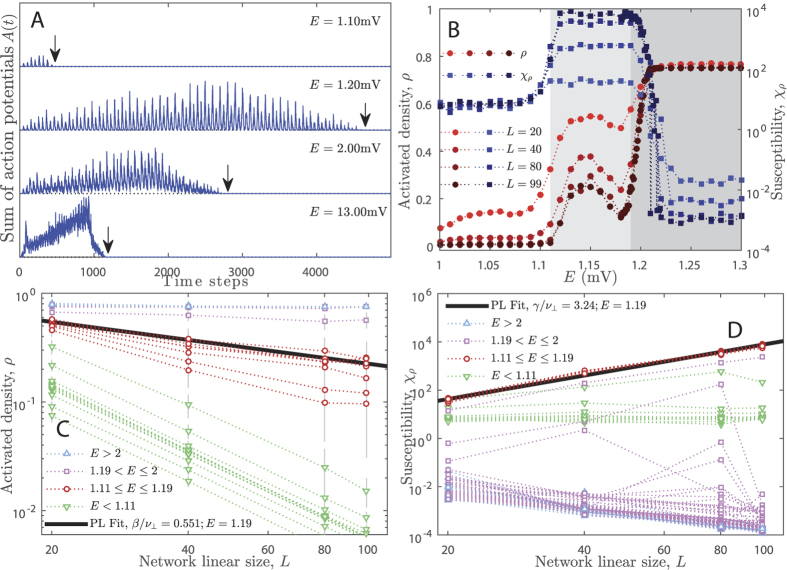
Network activity, order parameter and susceptibility. Panel A: Temporal profile of the avalanches for many EPSP. Arrows mark the processing time *T* of the network. For *E* = 1.1 mV, only very small avalanches occur; *E* = 1.2 mV shows many small avalanches; for *E* = 2.0 mV there is a dominating avalanche (compare to the dotted line that marks zero activity); and for *E* = 13.0 mV the dominating avalanche takes over all the dynamics. Panel B: Density of activated neurons *ρ* during total activity time and its associated susceptibility^55^ *χ*_*ρ*_ = *N*(〈*ρ*^2^〉 − 〈*ρ*〉^2^)/〈*ρ*〉 as function of *E* for many *L*. Red circles (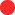, 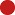, 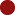, 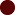) indicate *ρ* and blue squares (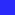, 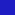, 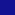, 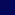) indicate *χ*_*ρ*_; the larger *L* the darker the color shade. White background indicate the inactive phase, light gray background indicates the critical (Griffiths) phase with diverging *χ*_*ρ*_ and dark gray background indicates the percolating phase. Panels C,D: FSS of *ρ* order parameter^26^,^28^ yielding scaling exponent *β*/*ν*_⊥_ = 0.55(3) – Equation (4) – and FSS of *χ*_*ρ*_ yielding scaling exponent *γ*/*ν*_⊥_ = 3.1(1) – Equation (5); fits performed on the transition point *E*_*c*_ = 1.19 mV; (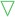) inactive phase, (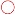) critical phase, (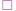) and (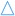) percolating phases. Vertical bars are standard deviation and dotted lines are only guides to the eyes.

In the present case, the extended body of each neuron (or site) and the disordered structure of the network generates a spreading pattern that works like a branching process^53^. Nevertheless, the adjusted *R* ensures that the activity will always die out and our model (as considered in this paper) has no active stationary state (see in Fig. 2A that activity eventually fades away for every *E*). After the activity of a single stimulation has ended, the amount of neurons that fired is *N*_*R*_. The configuration of the network made of *N*_*R*_ activated neurons and *N* − *N*_*R*_ inactivated neurons is an absorbing state. As we increase *E*, we notice that a larger fraction of neurons gets activated by the initial stimulus. For *E* > *E*_*th*_, the activity will definitely percolate the network before getting extinguished (i.e. *N*_*R*_ = *N*). Our model, thus, presents an absorbing state phase transition from a non-percolating inactive phase (small *E*) to a dynamically percolating phase (large *E*)^54^. We employ a mean-field analysis to determine *E*_*th*_ in the next subsection. Intermediary values of *E* yield vanishing non-zero probability of percolation.

After the activity is ceased, another trial is started by (a) rewiring the network according to the rules described in last section and in the Supplementary Material; (b) resetting the state of the neurons; and (c) flashing the same square stimulus to the network. This procedure is repeated many times for each *E* and *L*. Therefore, the averages and variances presented in this section are calculated over these several trials of the network quenched disorder in the connections between neurons. The disorder of the system creates rare region fluctuation effects on the size *N*_*R*_ of the percolation cluster for a finite range of the *E* parameter. The system then presents a GP in such interval^26^,^27^,^28^. Fig. 2A shows the temporal profile of activity *A*(*t*) (the sum of every soma spike in the network for a given time step) for a single trial of the network with *L* = 99 and four different *E*.

The temporal profile of activity comprises interspersing small and large peaks of activity that spontaneously emerge (see Fig. 2A and details in Fig. S2) due to the delay caused by the propagation of potentials through dendrites and axons. Thus, the separation of the peaks (i.e. the separation of *time scales*) in our model output data is not externally imposed, as commonly done in SOC models^22^. This emergent separation of activity intervals provides a natural way to define the avalanches of our model. The observed avalanches are correlated because the peaks follow from each other in an organized temporal sequence. This is different from the Poisson independently generated avalanches obtained in most models of absorbing state phase transitions^8^,^9^,^13^,^22^,^46^,^47^,^48^,^49^.

In such models, each avalanche is generated by a single stimulus and one avalanche is defined as all the activity between two inactive absorbing states of the system^23^,^22^. On the contrary, the dynamics of our model allows us to define avalanches using the experimental protocol: here, one avalanche is all the activity between two moments of soma silence in the network, even if the system did not reach its absorbing state during such silent period. In fact, when analyzing experimental data there is no guarantee that the system is in its inactive absorbing state between consecutive avalanches because background activity of the network is always neglected by applying *ad hoc* thresholds to the electrodes’ signals^12^,^13^,^14^,^16^. The size *s* of the avalanche is the sum of all the activity *A*(*t*) between two consecutive instants in which *A*(*t*) = 0. In fact, each *s* is the area under each of the peaks in Fig. 2A. The distribution of avalanche sizes *s* of our model will be discussed in the sections to follow.

Absorbing state phase transitions are commonly studied through the definition of two order parameters^21^: the density of active sites in the active stationary state (which defines the critical exponent *β*); or the percolation probability (which defines the critical exponent *β*′). The second one is most commonly used for systems without active stationary state. However, the density of activated sites by an initial stimulus (i.e. the density of sites that pertain to the percolating cluster, also known as debris density) is also used to describe phase transitions without active absorbing states^32^. Thus, we chose to study our systems’ phase transition using the density of activated neurons, *ρ* ≡ 〈*N*_*R*_/*N*〉, as our order parameter, because it may be directly measured for each quenched disorder configuration of the network. Notice that the average is taken over the many trials of the network for fixed *E* and *L*: for a given trial, a quantity *N*_*R*_ of neurons has been activated after the activity dies out; *ρ* is then the average of *N*_*R*_/*N*. The amount of activated neurons for each trial is simply calculated by 
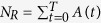
, where *T* is the total propagation time (time from the moment the activity is sparked until the moment it dies out). The quantity *T* is also known as mean survival time in absorbing phase transitions^21^.

The variance of *ρ* is regarded as a susceptibility, *χ *≡ *N*(〈*ρ*^2^〉 − 〈*ρ*〉^2^), and defines the critical exponent *γ*′ for models presenting typical absorbing phase transitions^21^,^55^. Nevertheless, notice that in such systems the variance of *ρ* is often taken over its temporal fluctuations in the absorbing active state^21^. Our model does not have an active absorbing state, so the fluctuations of *ρ* arise from different trials of the network disorder for fixed *E* and *L*, similarly to how *ρ* is measured: after each trial, a total density *N*_*R*_/*N* of activated neurons is left by the propagated network activity. Thus, the variance is calculated over all these trials for each *E* and *L*. Since our network is not regular, we chose to study a modified version of the susceptibility for complex networks, *χ*_*ρ*_ = *χ*/〈*ρ*〉, which defines a critical exponent^55^ *γ* = *γ*′ + *β*. Notice that this *γ* is not related to the average avalanche size of absorbing phase transitions. The standard susceptibility χ presents the diverging pattern with exponent *γ*′ expected for absorbing critical systems. However, the modified susceptibility shows in a more neatly way the extent of the GP.

Near the critical point, we may write the following scaling functions^19^,^21^,^28^,^26^:


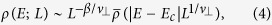



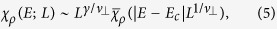


where *β, ν*_⊥_, and *γ* = *γ*′ + *β* are scaling exponents and 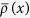 and 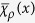 are universal scaling functions. If equations (4) and (5) hold for the computational data, then a critical phase transition occurs at *E* = *E*_*c*_^19^,^20^,^21^,^24^. The value *E*_*c*_ marks the point in which the probability of appearing a percolation cluster due to the system dynamics continuously changes from zero to positive^21^. This is the strong criterion to define criticality, although it is not commonly applied in neural networks models. Only one work has experimentally applied it so far in the brain context^25^. Notice that the exponent *γ*′ is calculated for the variance of *ρ* over the trials of the network, whereas the usual *γ*′ is obtained through temporal fluctuations. Thus, the *γ*′ we calculate in this work might not correspond to the usual *γ*′ of absorbing phase transitions.

We also compute the avalanche size distributions, 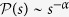, the complementary cumulative distributions, 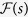, the avalanches’ autocorrelation, *C*(*t*′), and power spectrum, *S*(*f*), the activity time series DFA, *F*(Δ*t*), and measure the activity propagation time, *T*(*E*; *L*). In the critical point, these quantities may be written as:


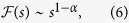



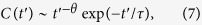



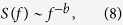



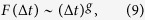



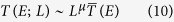


where *α* is the exponent of avalanche size distribution, *t*′ is the time lag between two avalanches, *τ* is the characteristic time of the autocorrelation exponential cutoff, *θ* and *b* are the autocorrelation and power spectrum exponents of the *avalanche time series*, respectively, *g* is the DFA exponent of the *activity time series, μ* is a scaling exponent and 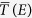 is a universal scaling function. Since *S*(*f*) is the Fourier transform of *C*(*t*′), *θ* + *b* = 1 for *t*′ ≪ *τ*.

The cumulative avalanche size distribution provides a clearer and direct way to calculate the cutoff *Z* of the avalanche size distribution. We assume that 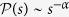 is valid for *s* ≤ *Z*, therefore


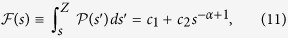


and thus *Z* = (−*c*_1_/*c*_2_)^1/(−*α* +1)^ with *c*_1_, *c*_2_ and *α* fitted to the cumulative distribution data^48^. At the critical point, the cutoff is expected to scale as *Z* ∼ *L*^*D*^ and *D* is an exponent defining a characteristic dimensionality of the avalanches^22^. If this scaling relation does not hold, then the system is not critical.

### Mean-field approximation

It is clear in Fig. 2A that there is a change in the activity profile as *E* is varied. The signal may reach only a few neurons of each layer for *E* = 1.1 mV whereas for 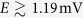, it excites the whole network. Such a behavior arises from the competition between excitation and dissipation. The excitation level of the network is controlled by the EPSP parameter, *E*, whereas the dissipation level is controlled by the attenuation constant, *λ*.

At the point where excitation balances dissipation, one expects that every neuron in the network will fire once. We will call this point the *activation threshold*, where *E* = *E*_*th*_. In order for any neuron *i* to fire, the signal that reaches the soma should be greater than or equal to the firing threshold, *v*_*T*_:


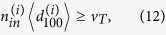


where 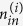 is the average amount of inward synapses per dendritic compartment of the postsynaptic neuron *i*, 
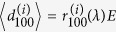
 is the average signal arriving on the soma compartment from one single synapse on any dendritic compartment of neuron *i* and 
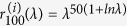
 is the fraction of the signal that reaches the soma as function of *λ* from a single presynaptic cell. See Supplementary Material for a derivation of 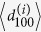.

Since the structure of the network is fixed for each trial, we may estimate 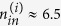 (from Table S2 in Supplementary Material). It is then simple to calculate *E*_*th*_ from Equation (12): 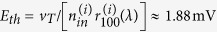, which we know from numerical data to be overestimated. Anyway, it is still close to the average experimental value of the *E* in the cortex *E* ≈1 mV^29^,^30^,^31^.

### Computational results

We performed between one hundred and three hundred trials of the network disorder for each EPSP of each *L* in order to calculate averages [equations (4) to (10), 10, 15, 16, 17, 18, 19] and standard deviations (vertical bars in Fig. 2).

#### Griffiths phase and critical phase transition

A critical point is found for *E* = *E*_*c*_ if^19^,^20^,^21^,^26^,^28^: (a) *ρ*(*E* = *E*_*c*_; *L*) → 0 according to Equation (4) *χ*_*ρ*_(*E* = *E*_*c*_; *L*) → +∞ according to Equation (5); both when *L* → ∞. It is easy to notice that both conditions are fulfilled within the light gray region in Fig. 2B (circles stand for *ρ* and squares stand for *χ*_*ρ*_). The light gray region is called a GP because it is an extended region that satisfies both conditions, instead of being a single critical point. This GP is due to the rewiring of the network, which originates rare regions. In some connection patterns the network allows neurons to fire more easily. Although rare, the presence of this behavior in the range of 1.11 ≤ *E* ≤ 1.19 causes large fluctuation of *ρ* when several trials of the network are considered.

In fact, Fig. 2C,D show that the PL scale functions [Equations (4) and (5)] are satisfied inside the whole EPSP range 1.11 ≤ *E* ≤ 1.19: the circles and squares in the light gray region in Fig. 2B correspond to the red circles in panels C and D. These panels explicitly show that *ρ* → 0 and *χ*_*ρ*_ → ∞ for *L* → ∞. We fitted these equations to the data on *E* = 1.19 mV ≡ *E*_*c*_ and obtained the critical exponents *β*/*ν*_⊥_ = 0.55(3), *γ*/*ν*_⊥_ = 3.1(2) and *γ*′/*ν*_⊥_ = (*γ* − *β*)/*ν*_⊥_ = 2.5(2). See the Supplementary Material for comments on the values of these exponents. Notice that *E*_*c*_ = 1.19 mV is of the same order of our naive mean-field calculation, *E*_*th*_ ≈ 1.88, although *E*_*c*_ is even closer to the average EPSP in the cortex *E* ≈1 mV^29^,^30^,^31^.

The left-hand side of the GP (white background in Fig. 2B) has *ρ* = 0 (green upside down triangles in Fig. 2C,D) and is named the inactive phase whereas the right-hand side (dark grey background) of the GP has *ρ* growing rapidly to saturated *ρ* ≈ 1 (purple squares and blue triangles in Fig. 2C,D) and is named the percolating phase. Both of these phases have finite susceptibility for increasing *L*.

#### Avalanche distributions

PL avalanche size and duration distributions are believed to be the ultimate signature of criticality in avalanching dynamical systems since the seminal work of Bak *et al*.^7^,^22^. However, recent works have shown that either critical systems may have no PL distributed avalanches^39^ or non-critical systems may have PL distributed avalanches^40^. We have shown in the previous section that our model has a continuous phase transition through a GP. Then, this section is devoted to show that PL avalanches may emerge in our system even outside of the critical regime. The scaling of the cutoff of the distributions must be calculated as function of the system size in order to have a better estimate of the critical regime of the system. In addition, we highlight some features of the avalanche distributions which are related to the structure of the network.

**Figure 3:**
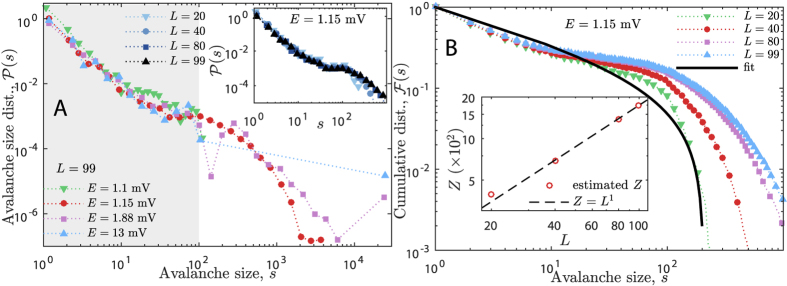
Avalanche size distributions and cumulative distributions. Panel A: Typical avalanche distributions 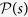 for many *E*: (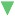) inactive phase, (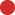) critical phase, (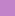) and (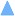) percolating phases. Note the bump around avalanches of size *N*_*c*_ ≈ 200 for *E* = 1.88 mV (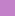); this bump reveals the internal structure of the network (see text for discussion). The light gray background highlights the range of avalanche size *s* in which all phases have PL-shaped distributions. Panel A inset: avalanche distributions for the critical phase (*E* = 1.15 mV) for increasing *L*. Panel B: avalanche cumulative distributions 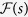 corresponding to panel A inset, *E* = 1.15 mV (the critical phase), for increasing *L*. Solid line is the fit of Equation (11) used to estimate *Z*(*L* = 20) and *α* = 1.4(1). Panel B inset: scaling law of *Z* ∼ *L*^*D*^ for *E* = 1.15 mV yielding *D* = 1.0(1); this scaling holds inside the whole critical phase. Avalanche distributions for the other phases have different *D* and are presented in Fig. 4.

Figure 3A shows the distribution of avalanche sizes *s* for *L* = 99 and four typical EPSP: *E* = 1.1 mV (inactive phase), *E* = 1.15 mV (GP), *E* = 1.88 mV (percolating phase), and *E* = 13 mV (percolating phase). Notice that all the four distributions have PL shape inside the highlighted region (*s* ≤ 100). We confirmed the PL decay of these distributions via the Maximum Likelihood test presented in the Supplementary Material. Therefore, a PL shape alone in the distribution of avalanche sizes is not enough to determine which distribution correspond to a critical regime. Sethna’s scaling law (a law which relates avalanche sizes and durations and is expected to hold only in criticality) also holds for the four phases of the model^53^.

The distribution of avalanche sizes for fixed *E* = 1.15 mV (inside the GP) is shown in the inset of Fig. 3A for systems of sizes *L* = 20, 40, 80, 99. Notice that the larger the system the farther the reach of the distribution. This fact becomes clearer in the plot of the cumulative distribution of *s* in Fig. 3B. Although the cumulative distributions seem to be turning flat for large *L*, they are actually accumulating towards a non-zero slope as *L* increases corresponding to a PL with exponent −*α* + 1. We fitted Equation (11) to the distributions in this figure in order to estimate their cutoffs, *Z*(*L*), and their PL exponent, *α* = 1.4(1) (this value is obtained for all system sizes). The plot of *Z*(*L*) is in the inset of Fig. 3B. We fitted *Z* ∼ *L*^*D*^ for the cutoffs and obtained *D* = 1.0(1). This value agrees with the scaling exponent for the largest avalanche of the system presented in the Supplementary Material.

**Figure 4:**
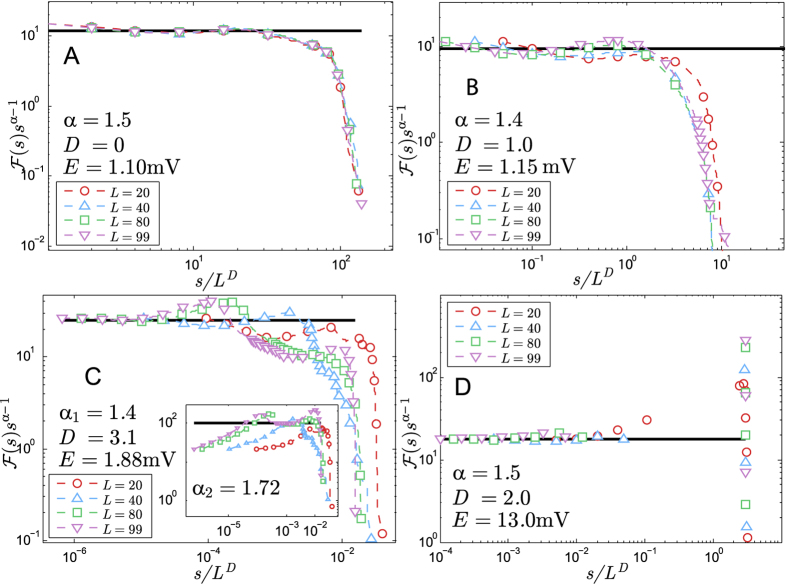
Collapse of avalanche size cumulative distributions for many realizations of the simulation for each *E*. Panel A: Collapse of avalanches in inactive phase; the cutoff does not scale with system size (*D* = 0) but the distribution presents a PL regime with exponent *α* = 1.5(1) due to noisy activity in the LGN. Panel B: Collapse of avalanches in Griffiths (critical) phase with exponents *α* = 1.4(1) and *D* = 1.0(1) corresponding to Fig. 3B. Panel C: Collapse of avalanches in weakly percolating phase with exponents *α*_1_ = 1.4(1), *α*_2_ = 1.72(8) and *D* = 3.1(3); the bump separating both PL ranges is a consequence of the columnar structure of the network, as it lies where *s* ≈ *N*_*c*_. Panel D: Collapse of avalanches in strongly percolating phase with exponents *α* = 1.5(1) and *D* = 2; the gap in this distribution shows that propagation occurs mainly through a large dominating avalanche, so the PL scaling represents only noisy avalanche activity in the LGN. The straight black lines guide the eyes over the collapse of the PLs. Notice that *α* is the exponent of 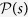. All the PL exponents were checked using Maximum Likelihood test (see Supplementary Material).

Figure 4 shows the collapse of the cumulative distributions, 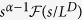 for many *L*. Each panel corresponds to a different phase of the model. Panel B of Fig. 4 corresponds to the collapsed data for *E* = 1.15 mV (inside the GP) yielding *α* = 1.4 and *D* = 1.0(1), evidencing the PL shape and the scaling of the cutoff of the cumulative distribution in the GP. Both values agree very well with the fitted data from Fig. 3B. The data corresponding to the inactive (or subcritical) phase is collapsed in Fig. 4A yielding *D* = 0 (avalanches do not scale with system size) and *α* = 1.5. The data corresponding to the percolating (or supercritical) phase is presented in panels C and D of Fig. 4. The exponent *D* varies inside this phase between *D* = 3 (for 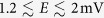) and *D* = 2 and becomes *D* = 2 for large *E*. The exponent *α* also varies and becomes *α* = 1.5 for large *E*.

The characteristic dimension *D* of avalanches sizes indicates how the activity spreads throughout the network^53^: *D* = 1 means that the activity is rather spreading through the columns of the model, *D* = 3 means that activity is spreading both radially and inside the columns and *D* = 2 means that the activity is spreading only radially and simultaneously within all the layers. Thus, the different values of *D* in the percolating phase give rise to two dynamically distinct phases: the weakly percolating regime (for 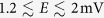) and the strongly percolating regime (for 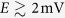). A detailed discussion concerning the spreading of the network activity is presented elsewhere^53^.

We identified two PL ranges for the avalanche distributions in the weakly percolating regime (see the purple squares in Fig. 3A and all the curves in Fig. 4C). The first range, *s*  < *N*_*c*_, has *α*_1_ = 1.4(1) and the second range, *s* > *N*_*c*_, has *α*_2_ = 1.7(1). *N*_*c*_ ≈ 200 is the amount of neurons in a single column of the network. These ranges are separated by a characteristic bump located at *s* ≈ *N*_*c*_ that is generated by the columnar structure of the network. If the column size, *l*, tended to *L*, the left-most bump would move to the right until merging with the right-most bump (the cutoff of the distribution). Then, there would be a single PL for the avalanches as the one presented by the columnless layered model of Teramae & Fukai^56^.

#### Long-range correlations and processing time

From Fig. 2A, it is clear that avalanches are temporally organized: each of the four presented time series has a low amplitude beginning, a growth until a maximum amplitude and then the activity decreases until fading out. We computed the sequence of avalanche sizes, *s*(*n*), its autocorrelation, *C*(*t*′), and its power spectrum, *S*(*f*). The time *t*′ is the lag between every two avalanches, *s*(*n*) and *s*(*n* − *t*′). The DFA is calculated over the raw time series, *A*(*t*), presented in Fig. 2A. These calculations are described in the Supplementary Material.

**Figure 5:**
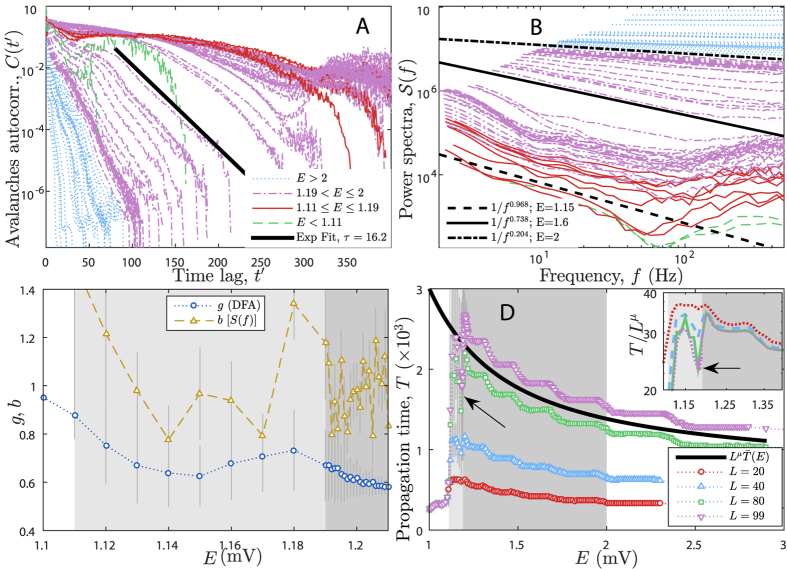
Autocorrelation, power spectrum, DFA and processing time. Panel A,B: Average autocorrelation (**A**) and average power spectrum (**B**) of avalanche sizes time series for *L*  = 99 and many *E*: (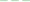) inactive phase, (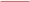) critical phase, (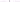) and (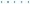) percolating phases. The bold lines show the exponential cutoff fit of the autocorrelation for *E* = 1.6 mV giving *τ* = 16.2 ts (A); and the fit *S*(*f*) ∼ *f*^*−b*^ giving *b* = 0.97(5) for *E* = 1.15 mV (critical phase), *b* = 0.74(4) for *E* = 1.6 mV and *b* = 0.20(2) for *E* = 2 mV (weakly percolating phase) as examples (**B**). Notice how the curves’ slope in panel B smoothly vary and yield *b* ≈ 1 inside the GP (see panel C too). Panel C: Power spectrum and DFA exponents versus *E* inside and close to the GP. *b* is the avalanches power spectrum exponent and *g* is the DFA exponent of the activity time series (of Fig. 2A). Notice that *b* ≈ 1 and 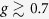 inside the critical region indicating long-range temporal correlations of both the avalanche time series and the spiking activity of the network. Panel D: Activity propagation time *T* as function of *E* for many *L*; solid line indicates the asymptotic behavior of *T*. Panel D inset: collapse plot 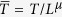 yielding *μ* = 0.9(1). Arrows indicate the minimum of *T*, vertical bars are standard deviation, light gray is the GP and dark gray is the weakly percolating phase. See Supplementary Material for more details about *T*.

The autocorrelation function of avalanche sizes is presented in Fig. 5A and the power spectrum of avalanche sizes is given in Fig. 5B. Each of the curves is averaged out of many realizations of the simulation. The more long-lasting correlations are inside the GP (red curves) or very close to it (purple curves for 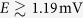). The characteristic time *τ* has been fitted by the exponential cutoff of Equation (7). *τ* is expected to scale with exp(*L*^*D*^) inside a GP^26^,^28^,^57^. We present the study of *τ* in the Supplementary Material.

The power spectrum shows a stable *f*^−*b*^ behavior with *b* ≈ 1 inside the critical phase (see Fig. 5B, red curves) for *f* < 100 Hz, suggesting long-range correlations in the avalanche size time series. In fact, the smooth change of slopes in Fig. 5B indicates that *b* varies continuously with *E* (green squares in Fig. 5C). We calculated the DFA exponent *g* for the activity time series in order to verify the long-range correlation. The exponent *g* also varies continuously with *E* (purple upside down triangles in Fig. 5C) and remains bounded in the interval 0.7 ≤ *g* ≤ 1 inside the GP, confirming the presence of long-range correlations. Both exponents *b* and *g* are close to the expected experimental values^33^,^35^,^36^.

Figure 2A also shows that the activity propagation time (also known as mean survival time in absorbing state phase transitions^21^) has a non-monotonic behavior with *E*. Fig. 5D shows in detail how *T* varies with *E*. We found that *T* has a deep local minimum at *E* = 1.18 mV (pointed by arrows in Fig. 5D) and a global maximum around *E* = 1.2 mV. The maximum grows with system size in the critical point *E*_*c*_ according to the law *T* ∼ *L*^*μ*^ [Equation (10)], as expected^21^. After that, *T* slowly decays asymptotically through a landscape full of shallow minima. The more intense the EPSP, the less active presynaptic neurons are needed to propagate the signal. As a result, the network as a whole will take less time to get activated for large *E*. The solid line is an arbitrary fit to the asymptotic decay of *T*(*E*). The FSS exponent of the propagation time is *μ* = 0.90(1) [see Equation (10) and Supplementary Material]. The rescaled propagation time, 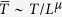, is shown in the inset of Fig. 5D.

For *E* < *E*_*c*_, *T* versus *E* is shaped similarly to *ρ* (compare dark circles in Fig. 2B to the behavior of *T* in Fig. 5D): the more neurons get activated, the more time the signal takes to completely cease. Such proportionality causes a deep local minimum in *T* near the phase transition and inside the critical phase. Additionally, *T* has maximum variance inside all the GP (see Supplementary Material), meaning that although the signal propagates quickly (in average), the system is flexible enough to adapt to external input as suggested by experimental work^16^. Therefore, it may be conjectured that the processing of information in V1 occurs preferably around this region.

## Concluding remarks

We studied a model for the visual cortex presenting its characteristic columnar structure. The key elements of this model are the neuron’s dynamical structure and dendritic excitation/dissipation balance. While the delay due to action potential propagation in the dendrites and axons causes the avalanche separation of time scales, the signal attenuation balances the network propagation of activity. If we were to model lateral inhibition inside the layers, we would expect the activation threshold *E*_*th*_ and the critical point *E*_*c*_ to grow in accordance with inhibition levels, leaving the described phase transition qualitatively unaltered. Certainly, the absorbing states of the system would depend on the reach, the quantity and the intensity of the lateral connections inside the layers. The detailed analysis of this scenario is a matter of future work.

We may summarize this work within five main findings:

- The extended critical phase is confirmed by the scaling laws of the density of activated neurons and of its associated susceptibility. The quenched disorder of the network generates the zero mean and the divergent variance of the density of activated neurons in an extended region of the parameter space. This phase is known as a Griffiths phase^26^,^28^. The critical phase lies near the average value of EPSP in the cortex (namely *E* ≈ 1 mV^29^,^30^,^31^).
- Avalanche size distributions are PL-shaped and their cutoff scale with system size as expected inside the critical region. However, avalanches also present a PL shape within a limited range of *s* for non-critical phases, corroborating the argument that PL avalanches alone (without the cutoff scaling law verified) are not necessarily connected to criticality^39^,^40^.
- The model displays long-range temporal correlations inside the critical region: the power spectrum of avalanche sizes have the form 1/*f *^*b*^ with *b* ≈ 1 whereas the activity time series DFA exponent is 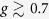; both match experimental results^17^,^33^,^35^,^36^.
- The model columnar structure was evidenced in the characteristic scale of small avalanches in the avalanches size distributions for the weakly ordered regime. We hypothesize that the two different power-law regimes could be experimentally found if the visual system was working in a slightly supercritical regime; for instance, with *E* = 1.88 mV.
- We discovered a local minimum of the network propagation time inside the Griffiths phase close to *E*_*c*_. At *E* = *E*_*c*_, the propagation time diverges as expected for absorbing phase transitions. Although minimum, the variance of the propagation time is maximum, resembling the variance of the order parameter. We conjecture that this behavior could be essential for a reliable processing of information in V1. Inside the inactive or strongly percolating phases, either the network will respond with noisy activity or will fire a quick single dominating avalanche.

All these evidences strongly suggests that visual processing occurs preferably at or near the critical phase. Our system’s observed criticality results from the average over the rewiring of the network quenched disorder. In the real brain, cortex synapses are reinforced or weakened on the time scales of seconds to hours, altering the microscopic structure of the network^58^. This phenomenon is known as sensory adaptation^59^. Moreover, the rewiring of brain connections occurs preferably during quiescent states, in which there is no processing of information^59^ – similarly, after the network has become inactive, we rewire it in order to stimulate the system again. Thus, GP could potentially be experimentally measured if one considers a long-term measurement of brain activity. Such data would naturally comprise different trials of connections for the propagation of activity in the many regions of the cortex. Hence, averages of the long-term activity could potentially be subject to rare region effects.

The studied model presents essential SOC features, such as the separation of time scales (which emerges naturally in the critical regime), PL avalanches that scale with system size, long-range temporal correlations and approximately 1/*f* power spectrum (inside the critical regime). Still, this model does not present an explicit mechanism of self-organization that would allow it to reach the critical state independently of its parameters. Anyhow, the real visual system may adapt to external stimuli and is indeed self-organized^16^,^58^. Besides, our model presents an extended region of critical behavior and gives us hints that these rare region effects might play a role in the real systems. If so, the critical state would be easier to achieve by evolutionary means due to its extended region in the parameter space.

It is not our aim in this work to determine the universality class of our model. Some issues need to be addressed in order to do that:

1. The dimensionality of our network is not trivially determined: although *N* ∼ *L*^2^, the connections are chosen randomly between neurons of adjacent layers within a very small and well determined excitatory field smaller than L^²^. Notice also that multiple interactions between the same neighbors happen. On the other hand, mean-field (random) networks have connections distributed all around the network without multiple interactions.
2. Our size exponents *α* and *D* are well determined but they rely on an unusual definition of avalanches: we define avalanches between two consecutive instants of soma silence whereas usually they are defined between two absorbing states of the system.
3. The dynamics of our model is somewhat similar to the general epidemic process (GEP) or generalized GEP^32^; however, *γ*′ is defined for our model but not for these systems, since they are not subjected to quenched disorder.

We stress that the strong way to describe critical phase transitions in the brain is to define an order parameter and its associated susceptibility and check in which range of parameters the first goes continuously to zero and the second diverges according to power laws, instead of only studying avalanche distributions or long-range correlations.

Some future work may focus on improving some of the model’s features. We can add disorder in *E*, background noise, lateral inhibition, synaptic dynamics or plasticity^16^,^46^,^47^ to model adaptability, and use different input stimuli. We also plan to make the excitatory field of each neuron change with depth.

Our study indicates that being critical or quasi-critical is advantageous for the brain sensory networks. Our network architecture could be further used to inspire the development of pattern recognition applications because of the short processing time inside the critical region. The high variability of activated density of neurons may enhance the sensitivity to different patterns, which may also aid in the pattern recognition tasks. Finally, we hope to provide here a kinematic framework for microscopic cortical modeling.

## Additional Information

**How to cite this article**: Girardi-Schappo, M. *et al*. Griffiths phase and long-range correlations in a biologically motivated visual cortex model. *Sci. Rep.* **6**, 29561; doi: 10.1038/srep29561 (2016).

## Supplementary Material

Supplementary Information
