# Supplementary material for: Griffiths phase and long-range correlations in a biologically motivated visual cortex model
Source: Sci Rep. 2016 Jul 20;6:29561. doi: 10.1038/srep29561 (PMC4951650; doi:10.1038/srep29561)
Supplement: Supplementary Information [file srep29561-s1.pdf]

Supplementary material: Griffiths phase and long-range correlations in a biologically motivated visual cortex model

M. Girardi-Schappo[1], G. S. Bortolotto[1], J. J. Gonsalves[1], L. T. Pinto[2], and M. H. R. Tragtenberg[1,*]

[1]Departamento de Física, Universidade Federal de Santa Catarina, 88040-900, Florianópolis, Santa Catarina, Brazil
[2]Departamento de Engenharia Química e Engenharia de Alimentos, Universidade Federal de Santa Catarina, 88040-900, Florianópolis, Santa Catarina, Brazil
[*]marcelotragtenberg@gmail.com

ABSTRACT

Activity in the brain propagates as waves of firing neurons, namely avalanches. These waves' size and duration distributions have been experimentally shown to display a stable power-law profile, long-range correlations and $1/f^b$ power spectrum *in vivo* and *in vitro*. We study an avalanching biologically motivated model of mammals visual cortex and find an extended critical-like region – a Griffiths phase – characterized by divergent susceptibility and zero order parameter. This phase lies close to the expected experimental value of the *excitatory postsynaptic potential* in the cortex suggesting that critical behavior may be found in the visual system. Avalanches are not perfectly power-law distributed, but it is possible to collapse the distributions and define a cutoff avalanche size that diverges as the network size is increased inside the critical region. The avalanches present long-range correlations and $1/f^b$ power spectrum, matching experiments. The phase transition is analytically determined by a mean-field approximation.

1 Network architecture

The model was initially proposed by Andreazza & Pinto.[1–3] The whole network has $N_{full} = 2N_{io} + N$ elements, where $N = 4L^2$ is the amount of neurons composing the internal layers (LGN, VI, IVCβ and II/III, each with L^2 neurons), $N_{io} = (10L)^2$ is the amount of elements in each of the Input/Output layers. An example of the look of the network is in figure S1A for $L = 5$. The architecture of the network is illustrated in figure S1B, where arrows point the direction of the connections towards which the signal is propagated.

Layers are stacked as shown in Figs. S1B,C. We can assign each element i with a spatial position $\vec{r}_i = (x_i, y_i, z_i)$. Each layer is labelled from $z_i = 0$ (Input) to $z_i = 5$ (Output). (x_i, y_i) refer to the position in a $L \times L$ square grid for $z_i = 1, 2, 3, 4$. When $z_i = 0$ and $z_i = 5$ the position (x_i, y_i) is for a square grid of size $10L \times 10L$. The elements of the Input layer are the photoreceptors of the retina. The elements of the Output layer are only the axonal terminals which would connect to dendrites in the secondary area of the visual cortex (V2). The elements of the remaining internal layers are neurons.

There is a matrix of 10×10 photoreceptors positioned in front of each neuron of the LGN (i.e. N_{io} photoreceptors). On the other end of the network, each neuron in layer II/III sends 100 synapses towards V2 (i.e. a total of N_{io} axonal terminals would connect to V2). The 100 axonal terminals of a given neuron are positioned in front of it, randomly distributed inside a 10×10 matrix.

All the connections are created in the beginning of the simulation (quenched disorder). The amount of connections that each element of a given layer attempts to send to any adjacent layer is in Table S1. Boundary conditions are free, which means that any attempted synapse that would fall outside of the ranges of the postsynaptic layer are discarded. Thus, the actual number of synapses per presynaptic element is a little smaller than the values in that table. They are similar to the numbers presented in Table S2.

Table S2 shows the typical amount of synapses that a given element of any layer receives for a single realization of the network The network is symmetric in average in the sense that the mean number of synapses per presynaptic neuron is almost equal to the mean number of synapses per postsynaptic neuron. Tables S1 and S2 also show the adjacency of layers.

In order to create a connection each presynaptic neuron, j, projects its axon towards a region of $l^2 = 7 \times 7$ neurons of an adjacent layer. The postsynaptic neurons in this region are then randomly selected via a bidimensional Gaussian distribution,

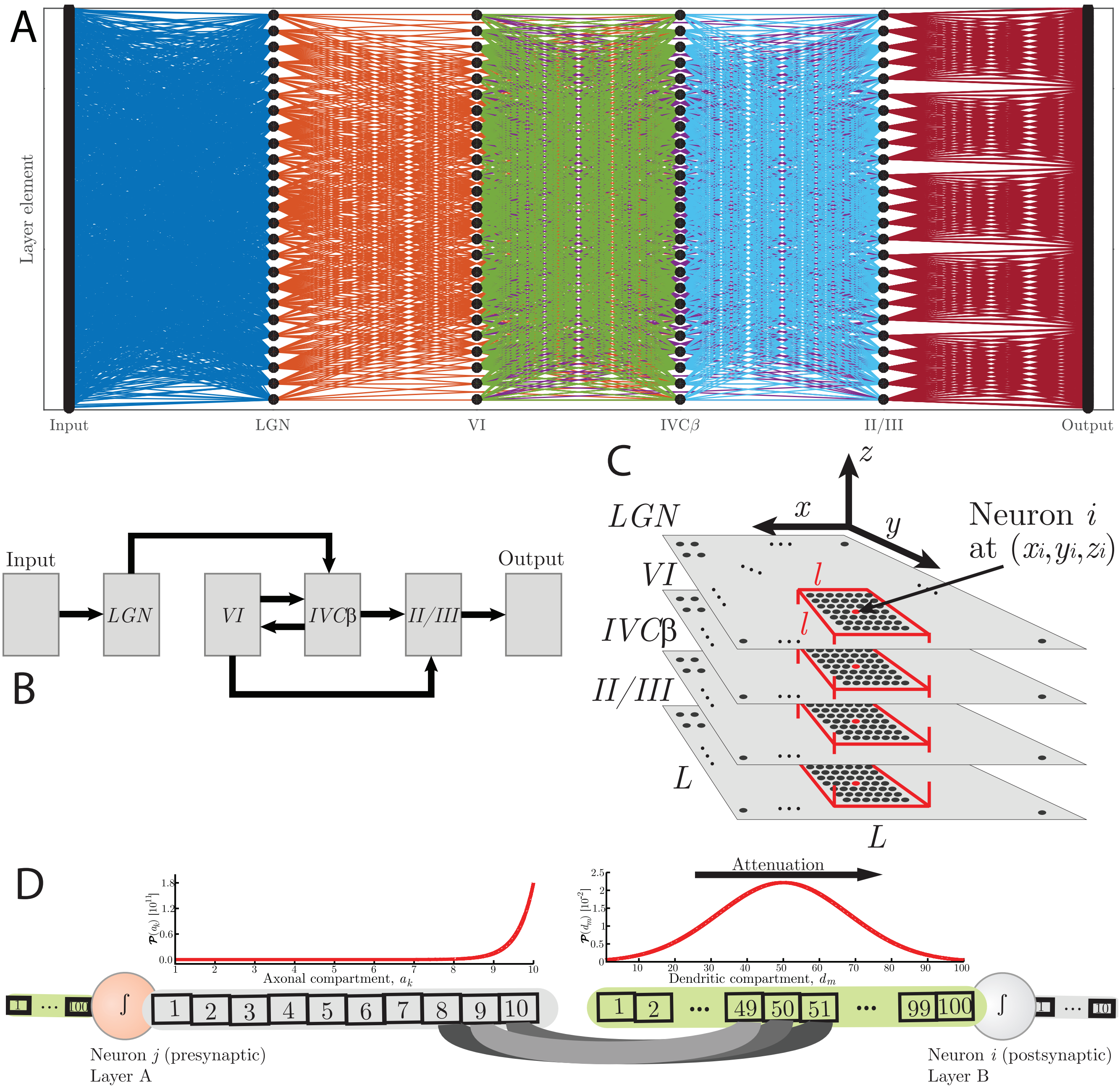

Figure S1. Elements of the V1 model. A: A realization of the $L=5$ network. Circles are photoreceptors (Input), neurons (internal layers) and axonal compartments (Output). Layers are linearized. B: Architecture of the network. C: Spatial organization of the network of $N=4L^2$ neurons. The columnar structure is highlighted in red. There is a column of size $N_c = 4l^2 = 196$ neurons centered on each neuron of the network. D: Compartmental scheme of neurons. The probability, $\mathscr{P}(a_k)$, of choosing a presynaptic axonal compartment, a_k, of any neuron is exponential such that most of the synapses start from the end of the axon (left). The probability, $\mathscr{P}(d_m)$, of choosing a dendritic postsynaptic compartment, d_m, is Gaussian with mean 50 and standard deviation 10, so that most of the synapses lay in the middle of the dendrite (right).

$\mathscr{P}_G(x,y;x_j,y_j,\sigma_d)$, centered in the (x_j,y_j) coordinates of the presynaptic neuron with standard deviation $\sigma_d = 3$ in each direction. The connections from Input layer to LGN are created using the same procedure.

A columnar structure emerges in the network as a consequence of picking neighbors in the limited region of l^2 neurons. V1 displays such structure as seen in experiments.[4] Therefore, each column has approximately $N_c = 4l^2 = 196$ neurons, see Fig. S1C. If one consider the loop between layers IVCβ and VI, then columns would virtually have $N_c = 5l^2 = 245$ neurons.

Once pre and postsynaptic neurons are chosen, synapses are created between one axonal compartment, $a_k^{(j)}$, chosen with

From \ To	LGN	VI	IVCβ	II/III	Output
**Input**	1	-	-	-	-
**LGN**	-	-	500	-	-
**VI**	-	-	1100	350	-
**IVCβ**	-	600	-	700	-
**II/III**	-	-	-	-	100

Table S1. The quantity of attempted synapses per presynaptic element from a specific layer (rows) to another layer (columns). Presynaptic element may be a photoreceptor (from Input layer) or a neuron (from internal layers). There are 100 photoreceptors in the Input layer for each neuron of the LGN. There are 100 axon compartments of each neuron in the II/III layer connecting to V2. These values are predefined in the beginning of the simulation, but are not exactly achieve in practice because of free boundary conditions.

From \ To	LGN	VI	IVCβ	II/III	Output
**Input**	98	-	-	-	-
**LGN**	-	-	488	-	-
**VI**	-	-	1073	293	-
**IVCβ**	-	585	-	683	-
**II/III**	-	-	-	-	1

Table S2. The quantity of synapses per postsynaptic element that a specific layer (column) recieves from another layer (row). Postsynaptic elements are all neurons. There are 100 axonal compartments in the output layer for each neuron and each of them forms a synapse with V2. The averages were performed when assembling a $L = 99$ network and are similar to the values from Table S1.

exponential probability $\mathscr{P}_E(k) = (10/4)\exp(10k/4)$, of the presynaptic cell, j, and one dendritic compartment, $d_m^{(i)}$, chosen with Gaussian probability $\mathscr{P}_G(m; d^{(c)}, \sigma_d)$, of the postsynaptic cell, i, where $d^{(c)} = 50$ and $\sigma_d = 10$. See figure S1D.

The probability of choosing a dendritic compartment is taken as Gaussian because consecutive events of connecting to any two compartments should be uncorrelated and most of the synapses should arrive around the center of the dendrites.[5] The probability of choosing an axonal compartment is exponential because axons should transport signals for as long as possible, thus most of the outward synapses must come from the end of the axon.[6–9]

The neurons of this model could be mapped into simple celular automata, like the one studied by Kinouchi & Copelli.[10] One should then adjust the probability of exciting a neighbor (in the referred model) as proportional to the fraction of the signal that would reach the soma in our model (derived in Section 3 of this Supplementary Material). The spike would have to be delayed in order to mimic the average amount of compartments in the neuron and the amount of refractory states would have to be adjusted accordingly in order to avoid self-sustained activity in the interlayer loops. Then, it is just a matter of building the structure of the network according to the rules presented in this section.

2 Macroscopic measurements

The *activity* $A(t)$ of the network at time t is just the sum of all neuronal firings at each time step,

$$A(t) = \sum_{i=1}^{N} \delta_{v_i(t),1} \ , \tag{1}$$

where $\delta_{i,j}$ is the Kronecker delta. The temporal profile of $A(t)$ is presented in Figure S2.

The density of activated neurons is simply

$$\rho = \frac{1}{N}\sum_{t=0}^{T} A(t) \ , \tag{2}$$

where T is the processing time of the network. Notice that ρ is already normalized as each neuron can only fire once.

The avalanche size, $s(n)$, is the sum of network activity,

$$s(n+1) = \sum_{t=t_n}^{t_{n+1}} A(t) \ , \tag{3}$$

where t_n is such that $A(t_n) = 0 \ \forall \ n$. Figure S2 (bottom) shows a detail of $A(t)$ for $E = 1.2$ mV. In this figure, we can see that $A(t)$ is zero for some time steps in between peaks. Such intervals are used to separate consecutive avalanches (i.e. consecutive peaks) in our data. The processing time is $T = \langle \max_n\{t_n\}\rangle$; the largest avalanche is $M = \langle \max_n\{s(n)\}\rangle$ (the average is taken across several trials for each E) and the ratio $m = M/N$ is the fractional largest avalanche.

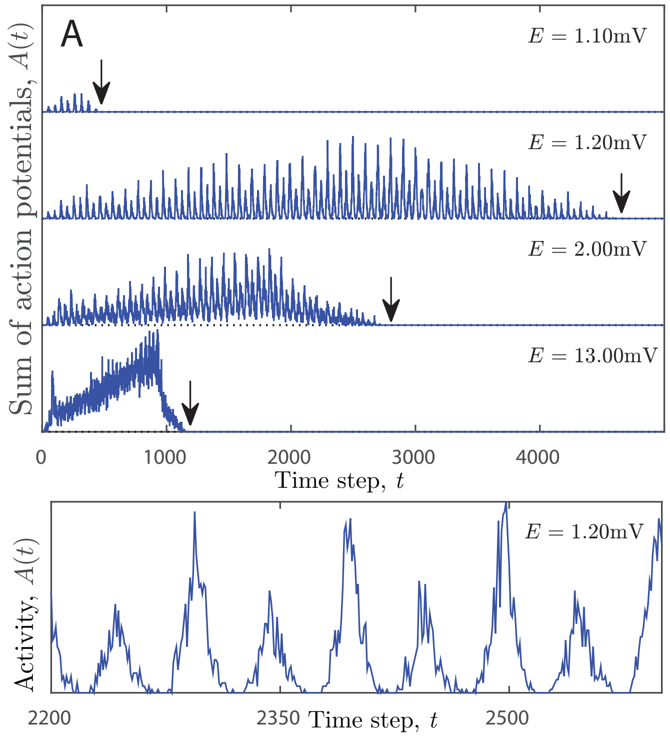

Figure S2. Temporal profile of activity, $A(t)$. Top: Temporal profile of the avalanches for many EPSP. Arrows mark the processing time T of the network. For $E=1.1$ mV, only very small avalanches occur; $E=1.2$ mV shows many small avalanches; for $E=2.0$ mV there is a dominating avalanche (compare to the dotted line that marks zero activity); and for $E=13.0$ mV the dominating avalanche takes over all the dynamics. Bottom: detail for $E=1.2$ mV in order to show the zeros of $A(t)$ used to separate different avalanches.

The avalanches autocorrelation function, $C(t')$, and power spectrum, $S(f)$, are defined as:

$$C(t') = \langle s(n)s(n-t')\rangle \ , \tag{4}$$

$$S(f) = \left| \int_{-\infty}^{+\infty} s(n)\exp(-2i\pi f n)\, dn \right|^2 \ . \tag{5}$$

The characteristic time, τ, of the autocorrelation function is defined by the exponential cutoff fit of $C(t') \sim \exp(-t'/\tau)$ for large t'. Moreover, if $S(f) \sim f^{-b}$ and $C(t') \sim t'^{-\theta}$ for small t', then $b+\theta = 1$ because $S(f)$ is the Fourier Transform of $C(t')$.

The *detrended fluctuation analysis* (DFA) is also a measure of long-range correlations for time series.[11–13] We apply this technique to the activity time series, $A(t)$, in order to obtain its fluctuations. First we obtain the average

$$\langle A\rangle = \frac{1}{T}\sum_{t=1}^{T} |A(t)| \ , \tag{6}$$

where T is the processing time. Then, we perform a cumulative sum over the detrended time series:

$$\bar{A}(t) = \sum_{t'=1}^{t} \left[A(t') - \langle A\rangle\right] \ , \tag{7}$$

for $t = 1, \cdots, T$.

We split the total time interval T in many boxes, each of size Δt. We then calculate the local trend of $\bar{A}(t)$ inside each window Δt simply by fitting a linear function to $\bar{A}(t)$ inside Δt. Let $y_{\Delta t}(t)$ be the y-coordinate of this fit, we may calculate the fluctuations by subtracting $\bar{A}(t)$ from each local trend inside each Δt window:

$$F(\Delta t) = \sqrt{\frac{1}{T}\sum_{t=1}^{T}\left[\bar{A}(t) - y_{\Delta t}(t)\right]} \ . \tag{8}$$

We repeat this procedure for several window sizes. $F(\Delta t)$ is expected to scale as:

$$F(\Delta t) \sim (\Delta t)^{g} \; . \tag{9}$$

The exponent g gives us information about the signal $A(t)$ autocorrelation:[11–13] $g < 1/2$: anti-correlated; $g \simeq 1/2$: uncorrelated, white noise; $g > 1/2$: correlated; $g \simeq 1$: $1/f$-noise, pink noise; $g > 1$: non-stationary, unbounded; $g \simeq 3/2$: Brownian noise.

3 Average signal in soma

Consider the structure described in Section 1 of this Supplementary Material for the dendrites of the neurons and the dynamics described in the main text. If a single synapse arrives at the compartment $d_n(0)$ of some neuron at instant $t = 0$, then after $k-n$ time steps the signal in dendrite $d_k(t = k-n)$ is $d_k = \lambda^{k-n}E$ with average $\langle d_k \rangle = \left\langle \lambda^{k-n} \right\rangle E$ (we omit time for simplicity).

The synapses arrive in any dendritic compartment, d_n, with Gaussian distribution $\mathscr{P}_G(n;50,10)$ with mean $d^{(c)} = 50$ and standard deviation $\sigma_d = 10$. Then, the average $\left\langle \lambda^{k-n} \right\rangle$ is given by:

$$\left\langle \lambda^{k-n} \right\rangle = \lambda^{k} \int_{-\infty}^{+\infty} \lambda^{-n} \mathscr{P}_G(n) dn \; , \tag{10}$$

where the integral is continuously evaluated within infinity limits because more than 99% of the Gaussian distribution lies within the bounds of the dendrite.

The integrand in Eq. 10 may be manipulated in order to still get a Gaussian function multiplied by a constant:

$$\left\langle \lambda^{k-n} \right\rangle = \lambda^{k-d^{(c)}+\sigma_d^2 \ln(\lambda)/2} \int_{-\infty}^{+\infty} \bar{\mathscr{P}}_G(n) dn \; , \tag{11}$$

$$\left\langle \lambda^{k-n} \right\rangle = \lambda^{k-d^{(c)}+\sigma_d^2 \ln(\lambda)/2} \tag{12}$$

where $\bar{\mathscr{P}}_G(n)$ is a normalized Gaussian with displaced mean. Notice that $r_k(\lambda) \equiv \left\langle \lambda^{k-n} \right\rangle = \langle d_k \rangle / E$ is the fraction of the signal that reaches compartment k.

If the synapse (or external signal) arrived just at compartment $d^{(c)}$ (i.e. $\sigma_d = 0$), then $r_k(\lambda) = \lambda^{k-d^{(c)}}$ would be a simple exponential decay with characteristic time $-1/\ln(\lambda)$, as experimentally expected.[5] In experiments the arriving signal compartment should be farther than 100 units from the soma. In our model the signal that reaches the soma is the signal from $k = 100$. The average signal in soma is therefore $\langle d_{100} \rangle = r_{100}E$.

4 Maximum Likelihood Estimation of Power Laws

We performed a Maximum Likelihood test in our avalanche distributions power-law fit. The columnar structure of the model is responsible for the presence of a *bump* in the shape of the obtained avalanche distributions. To take this into account we used a censored Maximum Likelihood estimator which uses only the data that exhibits a power law in order to calculate the best fit exponent, $\hat{\alpha}$. The formula follows the description for discrete power laws made by:[14]

$$\hat{\alpha} = \underset{\alpha}{\operatorname{argmax}}[-n \ln \zeta(\alpha, s_{min}, s_{max}) + \mathscr{H}], \tag{13}$$

$$\mathscr{H} = \sum_{i=1}^{n} \begin{cases} \ln\left(\sum_{k=s_{min}}^{s_L} k^{-\alpha}\right) & \text{for } s_i = s_L \; , \\ \ln\left(\sum_{k=s_R}^{s_{max}} k^{-\alpha}\right) & \text{for } s_i = s_R \; , \\ \ln\left({s_i}^{-\alpha}\right) & \text{else} \end{cases} \tag{14}$$

where $\hat{\alpha}$ is the expected value of the exponent of the best fit power law, $s \geq s_{min} \geq 1$ and the Hurwitz zeta function is defined by

$$\zeta(\alpha, s_{min}, s_{max}) = \zeta(\alpha, s_{min}) - \zeta(\alpha, s_{max}) \; , \tag{15}$$

where

$$\zeta(\alpha, s) = \sum_{i=1}^{\infty} \frac{1}{(i+s)^{\alpha}} \; . \tag{16}$$

We applied this test to the complementary cumulative distributions for some E values and $L = 99$. For $E = 1.10$ mV, $E = 1.19$ mV (critical point) and $E = 13.0$ mV we obtained best fit slopes with values $\hat{\alpha} = 1.46$, $\hat{\alpha} = 1.34$ and $\hat{\alpha} = 1.50$, respectively. For $E = 1.88$ mV we divided the distribution in two parts in order to avoid the bump around avalanches of size $N_c \approx 200$. For the first part (with $s_{min} = 1$, $s_{max} = 190$) we obtained a slope with value $\hat{\alpha}_1 = 1.69$ and for the second one (considering $s_{min} = 600$, $s_{max} = 1500$) we obtained the value $\hat{\alpha}_2 = 1.57$. All these values are fairly close to the values obtained by the avalanche cumulative distribution collapse and $\mathscr{F}(s) = c_1 + c_2 s^{-\alpha+1}$ fit presented in the main text.

5 Finite-Size Scaling of the fractional largest avalanche

The fractional largest avalanche (or cluster), m, is sometimes regarded as an order parameter to study the Griffiths phase.[15–18] In Figure S3 we show m as function of E for increasing L and the FSS of m for several E.

Inside the critical region we expect that $m \sim L^{-D'}$ as it is a possible order parameter. Indeed, our fit for $1.11 \le E \le 1.19$ mV (inside the Griffiths phase) gives $D' = 1.0(1)$. This value allows us to calculate the scaling of the largest avalanche (or cluster), M, because $m \equiv M/N$ and $N = 4L^2$. Therefore,

$$M = m(4L^2)$$
$$\Rightarrow M \sim L^{-D'} L^2 \,,$$

gives

$$M \sim L^{2-D'} \,. \tag{17}$$

Now, $D' = 1.0(1)$ and then $M \sim L^1$ which agrees with the avalanche cutoff exponent $Z \sim L^D$ with $D = 1.0(1)$ presented in the main text. $M \sim L^D$ is also expected inside the Griffiths phase.[15–17] The scaling law in Equation (17) is valid only at the critical point. Both exponents, D' and D, were independently fitted so this calculation supports the evidence for criticality.

6 Finite-Size Scaling of autocorrelation characteristic time

A widely used criterion to determine whether the system presents a Griffiths phase is the FSS of the autocorrelation characteristic time, τ, of the avalanche (or cluster) time series.[15–17] A system with a single critical point and no Griffiths phase is expected to have τ scaling as

$$\tau \sim L^z \,, \tag{18}$$

where L is system size and z is known as the dynamical exponent and is generally non-universal.[19] However, inside a Griffiths phase, τ behaves as a stretched exponential:[15–17]

$$\tau \sim \exp\left(cL^D\right) \,, \tag{19}$$

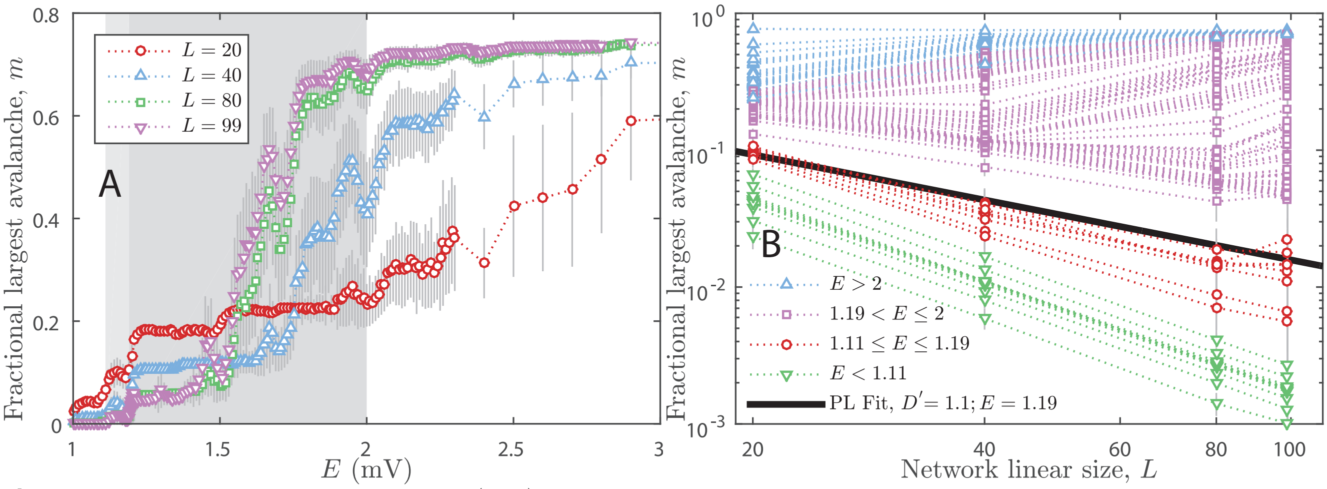

Figure S3. Fractional largest avalanche, $m(E;L)$. Panel A: m as function of E for many L. Panel B: m FSS. The red circles correspond to the critical (Griffiths) region. The fit $m \sim L^{-D'}$ yields $D' = 1.0(1)$ inside the Griffiths region.

where c is just a constant and D is expected to be the exponent of the largest avalanche defining a typical dimensionality of the system.

Figure S4 shows the same simulation data for $\tau(L)$ fitted by either the stretched exponential of Equation (19) (panel A) or the power law of Equation (18) (panel B). We ignored $\tau(L=20)$ for fitting the stretched exponential. Notice that both panels present plausible fits, although the stretched exponential fit yields $D = 1.1(1)$ which matches our expectations from other independent calculations of D (from the avalanches cutoff fit, Z, presented in the main text and from the largest fractional avalanche fit, m, presented in the previous section).

7 Processing time

The network processing time has been discussed in the main text and is again presented in Figure S5A for completeness. The FSS of $T \sim L^{\mu}$ is fitted in Figure S5B and gives $\mu = 0.80(1)$. The variance of T is defined as:

$$\mathrm{Var}(T) \equiv \left\langle T^2 \right\rangle - \langle T \rangle^2 \ . \tag{20}$$

Inside the Griffiths region we verified that $\mathrm{Var}(T)$ behaves as:

$$\mathrm{Var}(T) \sim L^{\mu'} \ . \tag{21}$$

We present $\mathrm{Var}(T)$ as function of E in Figure S5C and its FSS fit, Figure S5D, yields $\mu' = 3.5(2)$. There is a striking similarity between $\mathrm{Var}(T)$ and $\chi_\rho \equiv N\mathrm{Var}(\rho)/\langle \rho \rangle$, specially concerning the exponent values (μ' and $\gamma/\nu_\perp$).

For $E > E_c = 1.19$ mV and every L the processing time asymptotic behavior may be fitted by a crossover of two PL (see Figure S5A):

$$\bar{T}(E) \sim \left(E^{-\omega} + E^{-1/\omega}\right) \ , \tag{22}$$

where $\omega = 3.1(2)$. The term $E^{-1/\omega}$ dominates for $E \gg E_c$. This equation is empirically obtained and has no underlying principle. However, it was useful to estimate the simulation time. The understanding of the functional form in equation (22) will be considered in future works. Notice that the dependence on L is explicitly given in the Results section of the main text.

8 Small size effects on the calculations

Ignoring small sized networks simulation data points for fitting is generally acceptable due to finite-size effects (i.e. boundary effects) that are enhanced in small systems. In our case, each column in a network with $L = 20$ includes a fraction of more than 12% of all neurons in the layers. This is significantly different from fractions of other columns in networks with different L: 3% for $L = 40$ and less than 1% for $L = 80, 99$. Avalanches thus are able to grow larger and faster for networks with $L = 20$ than expected for other L with fixed E.

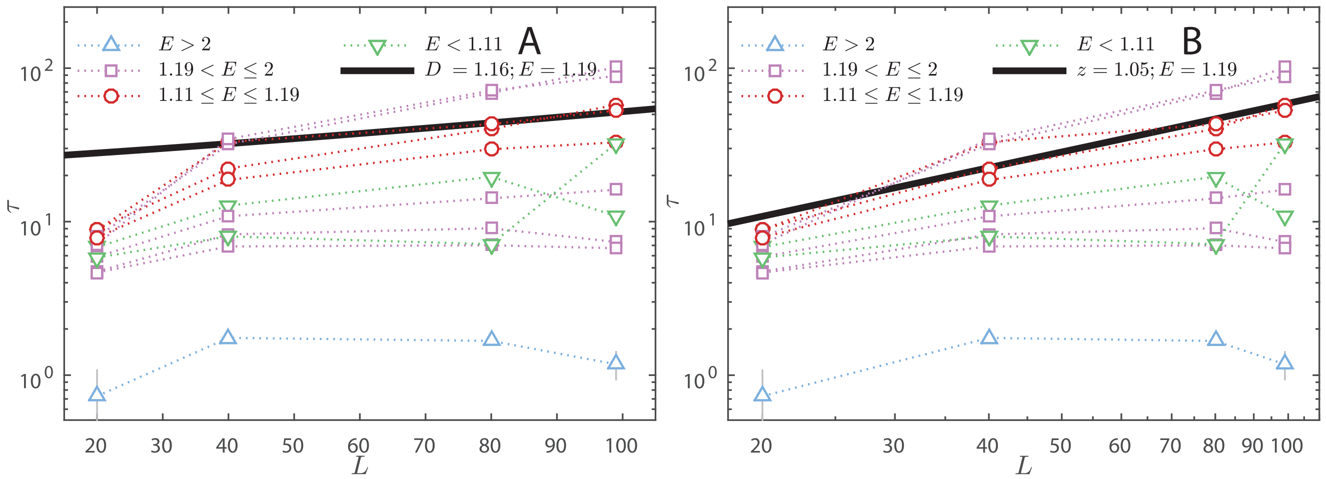

Figure S4. Autocorrelation characteristic time FSS. Panel A: stretched exponential fit [Equation (19)] as expected for a Griffiths phase; the fitted D agrees very well with the largest avalanche exponent calculated in the previous section, as expected. $\tau(L=20)$ was not considered for fitting. Panel B: power-law fit [Equation (18)] (considering $L = 20$) yielding dynamical exponent $z = 1.0(1)$.

Also, initial conditions are a flash of a fixed size square of size 30×30 in the Input layer of the model, corresponding to an average activation of $3 \times 3 = 9$ neurons in the LGN layer. Thus, an average fraction of $9/400 \approx 2 \times 10^{-2}$ of the LGN neurons are initially activated for $L = 20$. For larger L we have average fractions of 10^{-3} or less neurons of the LGN which are initially activated.

The case $L = 20$ would have the largest avalanche and the activated density slightly larger than expected. On the other hand, the variance of such order parameters has slightly lower values. $L = 20$ would also present processing time slightly lower than expected because the columns of such network are larger fraction of its layers. If this fact is taken into account and we ignore $L = 20$ in the FSS analysis of ρ, χ_ρ and Var(T), the exponents $\beta/\nu_\perp$, $\gamma/\nu_\perp$ and μ' significantly change from the values presented in the main text and in the previous section to $\beta/\nu_\perp = 0.40(2)$, $\gamma/\nu_\perp = 2.6(2)$ and $\mu' = 2.9(2)$. The scaling exponent of T, remains practically unchanged $\mu = 0.81(1)$ with or without $L = 20$. However, only the universality class of the model is changed. The phase transition is still a continuous phase transition, such that there is still a Griffiths phase in the very same region of EPSP, $1.11 \le E \le 1.19$ mV.

The best approach to tackle this problem would be to estimate the deviations caused by the small-size effects on $L = 20$ network and correct its corresponding data points, instead of deliberately ignoring them for fitting. Nevertheless, we do not have a systematic way of accomplishing this, so we are not yet able to definitively determine the model's universality class.

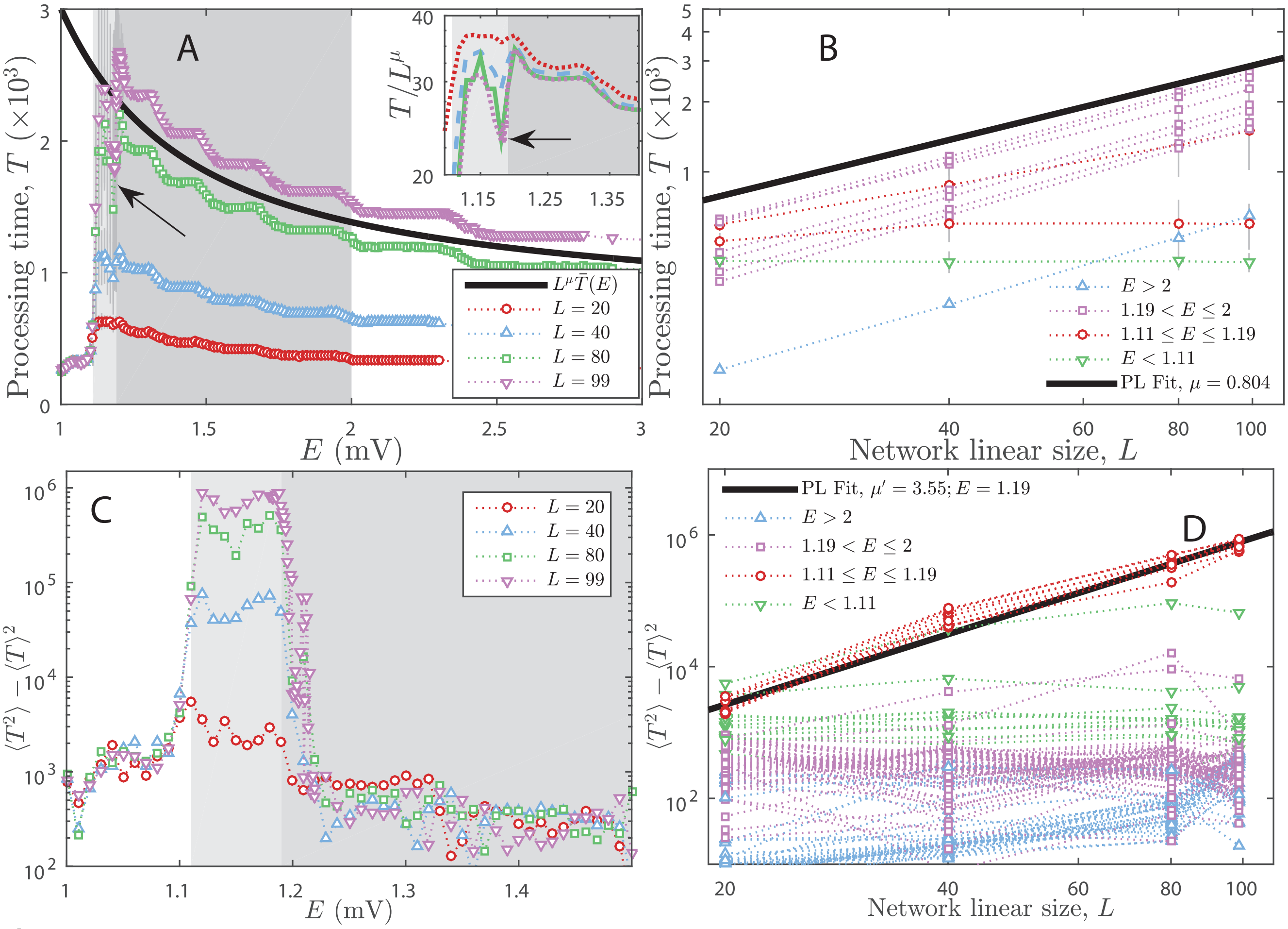

Figure S5. Processing time. Panel A: Processing time T as function of E for many L; solid line indicates the asymptotic behavior of T for $L = 99$ [Equation (22) fit with $\omega = 3.1(2)$]. Panel A inset: collapse plot $\bar{T} = T/L^\mu$ yielding $\mu = 1.0(1)$. Arrows indicate the minimum of T. Panel B: T FSS yielding $\mu = 0.80(1)$. This scaling is obeyed for $E \ge E_c = 1.19$. Panel C: Variance of the processing time [Equation (20)], Var(T), as function of E for many L. Panel D: FSS of Var(T), Equation (21), yielding $\mu' = 3.5(2)$. Notice the similarity between the FSS of χ_ρ (which is the variance of ρ) and Var(T). (▽) inactive phase, (○) critical phase, (□) and (△) active phases. Vertical bars are standard deviation, light gray is the Griffiths phase, dark gray is the weakly active phase and dotted lines are only guides to the eyes.

References

1. Andreazza, J. K. & Pinto, L. T. Simulation of the primary visual cortex of the macaque monkey by natural neural networks. In *Prooceedings of 2nd LNCC Meeting on Computational Modelling* (Petrópolis, RJ, Brazil, 2006).
2. Andreazza, J. K. & Pinto, L. T. Proposition of a physiologically plausible neuronalnetwork for the simulation of the primary visual cortex of the macaque monkey. In *Prooceedings of BICS 2008 – Brain Inspired Cognitive Systems* (São Luís, MA, Brazil, 2008).
3. Pinto, L. T. & Andreazza, J. K. The excitatory post synaptic potential and the dendrite space constant influences in the behaviour of system of neurons. In *Prooceedings of BICS 2008 – Brain Inspired Cognitive Systems* (São Luís, MA, Brazil, 2008).
4. Albright, T. D., Jessell, T. M., Kandel, E. R. & Posner, M. I. Neural science: A century of progress and the mysteries that remain. *Neuron* **25**, S1–S55 DOI 10.1016/S0896-6273(00)80912-5 (2000).
5. Williams, S. R. & Stuart, G. J. Dependence of EPSP efficacy on synapse location in neocortical pyramidal neurons. *Science* **295**, 1907–1910 (2002).
6. O'Kusky, J. & Colonnier, M. A laminar analysis of the number of neurons, glia and synapses in the visual cortex (area 17) of adult macaque monkeys. *J. Comp. Neurol.* **210**, 278–290 (1982).
7. Lund, J. S. Spiny stellate neurons. In Peters, A. & Jones, E. G. (eds.) *The Cerebral Cortex*, vol. 1, chap. 7, 255–308 (Plenum Press, New York, USA, 1984).
8. Callaway, E. M. Local circuits in primary visual cortex of the macaque monkey. *Annu. Rev. Neurosci.* **21(2)**, 47–74 (1998).
9. Yabuta, N. H. & Callaway, E. M. Functional streams and local connections of layer 4c neurons in primary visual cortex of the macaque monkey. *J. Neurosci.* **18(22)**, 9489–9499 (1998).
10. Kinouchi, O. & Copelli, M. Optimal dynamical range of excitable networks at criticality. *Nat. Phys.* **2**, 348–351 (2006).
11. Peng, C.-K., Havlin, S., Stanley, H. E. & Goldberger, A. L. Quantification of scaling exponents and crossover phenomena in nonstationary heartbeat time series. *Chaos* **5**, 82–87 DOI http://dx.doi.org/10.1063/1.166141 (1995).
12. Linkenkaer-Hansen, K., Nikouline, V. V., Palva, J. M. & Ilmoniemi, R. J. Long-range temporal correlations and scaling behavior in human brain oscillations. *J. Neurosci.* **21(4)**, 1370–1377 (2001).
13. Hardstone, R. *et al.* Detrended fluctuation analysis: a scale-free view on neuronal oscillations. *Front. Physiol.* **3**, 450 DOI 10.3389/fphys.2012.00450 (2012).
14. Langlois, D., Cousineau, D. & Thivierge, J.-P. Maximum likelihood estimators for truncated and censored power-law distributions show how neuronal avalanches may be misevaluated. *Phys. Rev. E* **89**, 012709 DOI 10.1103/PhysRevE.89.012709 (2014).
15. Bray, A. J. & Rodgers, G. J. Diffusion in a sparsely connected space: A model for glassy relaxation. *Phys. Rev. B* **38(16)**, 11461–11470 (1988).
16. Vojta, T. Rare region effects at classical, quantum and nonequilibrium phase transitions. *J. Phys. A: Math. Gen.* **39**, R143 DOI 10.1088/0305-4470/39/22/R01 (2006).
17. Moretti, P. & Muñoz, M. A. Griffiths phases and the stretching of criticality in brain networks. *Nat. Commun.* **4**, 2521 DOI 10.1038/ncomms3521 (2013).
18. Haimovici, A., Tagliazucchi, E., Balenzuela, P. & Chialvo, D. R. Brain organization into resting state networks emerges at criticality on a model of the human connectome. *Phys. Rev. Lett.* **110**, 178101 (2013).
19. Ódor, G. Universality classes in nonequilibrium lattice systems. *Rev. Mod. Phys.* **76(3)**, 663–724 (2004).
